# Diversity and environmental drivers of *Suillus* communities in *Pinus sylvestris* var. *mongolica* forests of Inner Mongolia

**DOI:** 10.1515/biol-2025-1156

**Published:** 2025-08-18

**Authors:** Rui-xia Liu, You-han Wu, Cong Li, Yi-hua Qiao, Yi-wen Yang, Wei-ping Yan, Qing-zhi Yao

**Affiliations:** College of Grassland Science, Inner Mongolia Agricultural University, No. 29 Ordos East Street, Saihan District, Hohhot, 010018, China; Taiwei Ecological Research Institute, Inner Mongolia Taiwei Ecological Technology Co., Ltd., Hohhot, 010200, China; College of Life Sciences, Inner Mongolia Agricultural University, Hohhot, 010018, China; Comprehensive Security Center, Ejin Banner Forestry and Grassland Bureau, Ejin Banner, 735400, China; Grassland Research Institute, Inner Mongolia Academy of Forestry Science, Hohhot, 010010, China; Ituri River Forestry Limited Liability Company, Yakeshi, 022150, China

**Keywords:** *P. sylvestris* var. *mongolic*a, soil fungi, *Suillus*, ectomycorrhizae

## Abstract

This study investigates the diversity and distribution of *Suillus* fungi in *Pinus sylvestris* var. *mongolica* (PSM) forests across Inner Mongolia, with a focus on understanding the environmental factors influencing fungal communities. High-throughput sequencing was utilized to analyze soil fungal communities across 12 PSM forest sites, alongside assessments of meteorological variables and soil enzyme activities. Thirteen *Suillus* species were identified, with *S. clintonianus* being the dominant species. The diversity of *Suillus* fungi exhibited significant geographical variation, with diversity decreasing from east to west. Precipitation and leucine aminopeptidase activity were identified as key drivers of fungal distribution. The soil fungal community was predominantly saprotrophic, playing a crucial role in nutrient cycling and ecosystem stability. The findings provide a deeper understanding of the role of ectomycorrhizal fungi in sustaining forest health and offer valuable insights for sustainable forest management and restoration efforts in semi-arid regions.

## 1 Introduction

*Pinus sylvestris* var. *mongolica* L. (referred to as PSM) is the primary evergreen species in the sandy regions of northern China, playing a crucial ecological role in windbreak and sand fixation. PSM is pivotal in ecological restoration and engineering projects across northern China, with its plantations extending over 700,000 hectares across more than 13 provinces in semi-arid areas [1]. These forests are vital for windbreak, soil structure improvement, and biodiversity enhancement. However, due to the impacts of climate change, overexploitation, and poor management, these forests are facing severe degradation, manifested by slow growth, sparse stands, and declining biodiversity. Soil moisture and nutrients are the primary ecological constraints limiting vegetation growth [2], and while soil microorganisms are a crucial component of forest ecosystems, in-depth studies on their impact on PSM growth and the mechanisms of forest degradation are lacking. Since the 1970s, extensive PSM plantations have become an integral part of the “Three-North” Shelter Forest Program. Although research has primarily focused on water factors, the degradation issues of PSM plantations are complex, involving physiological mechanisms such as hydraulic failure and carbon starvation [3].

Fungi are one of the major groups of plant-associated microorganisms, crucial for regulating plant health, maintaining interactions between plants and other organisms, and the overall functionality of ecosystems [4]. The functional interactions between trees and fungi are essential for trees to adapt to changing environments. Different fungal species and functional groups respond differently to environmental changes, driven by climatic, nutritional, and biological factors. Global multivariate analyses indicate that forest degradation leads to reduced soil carbon and nitrogen levels, increased soil pH, and accelerated carbon decomposition rates. Additionally, soil fungal biomass decreases at disturbed sites, but species diversity increases, closely correlating soil pH changes with shifts in fungal community composition [5]. In forest ecosystems, ectomycorrhizal (ECM) fungi hold a pivotal position, with suilloid fungi (i.e., the genera *Suillus* and *Rhizopogon*) exhibiting high host specificity with Pinaceae hosts [6]. Among ECM fungi, the genus *Suillus* is a pioneer species widely distributed in coniferous forests. *Suillus* comprises about 100 species and is primarily associated with the Pinaceae family, forming ECM relationships with conifers. These species are widely distributed across the northern hemisphere, with notable populations in boreal, temperate, and semi-arid ecosystems. *Suillus* species exhibit high host specificity, often forming symbioses with particular genera, subgenera, or species within the Pinaceae family. In semi-arid regions, such as the PSM forests of Inner Mongolia, *Suillus* species have developed unique ecological adaptations to cope with water scarcity and nutrient-poor soils. In these environments, ECM fungi like *Suillus* enhance nutrient uptake for host plants, particularly under conditions of water stress, making them vital for the survival and health of forests in arid and semi-arid regions. *Suillus* species are found in diverse ecosystems across the northern hemisphere, from boreal forests in Canada and Russia to temperate forests in Europe and North America. In semi-arid regions, such as the Mediterranean and parts of the western United States, studies have shown that *Suillus* species exhibit remarkable resilience to water stress and can thrive in ecosystems characterized by low moisture availability. Ecologically, *Suillus* is critically important as an underground partner for many Pinaceae in the northern hemisphere, often serving as a pioneer species of ECM fungi in northern afforestation and nursery practices [7]. Moreover, some *Suillus* species are edible and have been found to possess anticancer properties, making them suitable for medicinal uses [8–10].

Despite the significant ecological role of PSM in the Inner Mongolia Autonomous Region, research on soil fungi and the community composition of the genus *Suillus* in these forest ecosystems remains very limited. In light of this, the present study employs high-throughput sequencing techniques to thoroughly analyze the composition, distribution patterns, and diversity of soil fungal communities, particularly *Suillus* fungi, in typical PSM areas in Inner Mongolia.

The study aims to address the following research questions: What environmental factors most strongly influence the diversity and distribution of *Suillus* fungi in PSM forests? How do changes in environmental conditions, particularly precipitation and soil enzyme activity, affect *Suillus* community composition and forest ecosystem functioning in semi-arid regions?

## 2 Materials and methods

### 2.1 Sampling site overview

**Figure 1:**
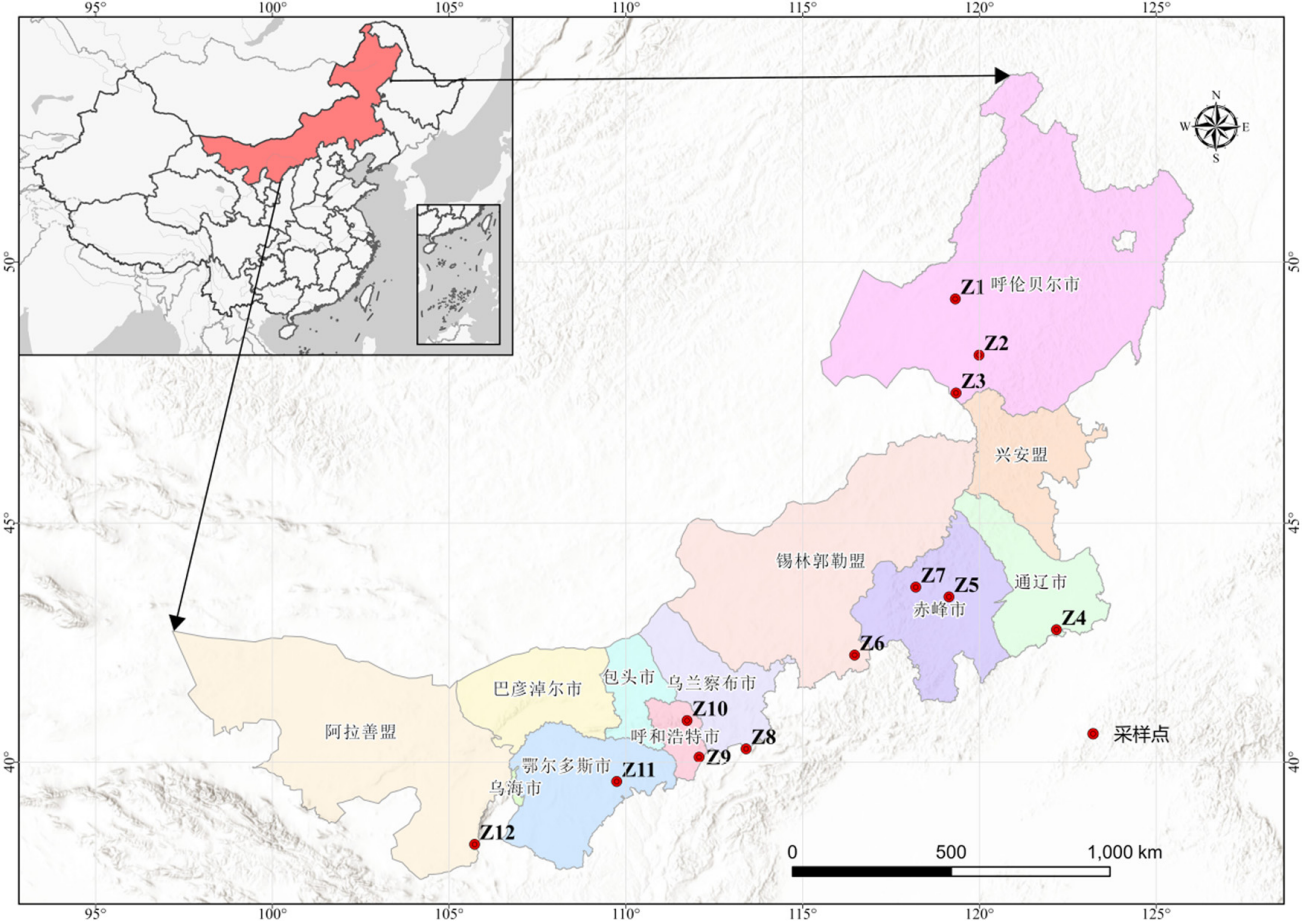
A map of sampling sites.

The experimental samples for this study were collected between August and September 2021. Twelve typical PSM forest areas were strategically selected across Inner Mongolia, representing a gradient of environmental conditions such as temperature, precipitation, and soil properties. These sites were chosen to capture the diversity of ecological conditions within the PSM forest ecosystem. Specifically, sites were selected based on variations in annual precipitation and temperature, as well as differences in forest degradation levels, ranging from well-preserved to degraded areas. This selection aimed to provide a comprehensive representation of fungal community diversity across varying environmental gradients within the semi-arid region. Data on the annual average temperature and annual mean precipitation (AP) were obtained from meteorological stations located at each of the sampling sites within the various leagues and cities of the Inner Mongolia region. Information on the sampling sites is presented in Table 1 and Figure 1.

**Table 1:** Overview of sampling site

Sample site	Location	Annual temperature (°C)	Annual precipitation (mm)
Hailar National Forest Park (Z1)	119.32°N 49.32°E	−1.5	331.4
Honghuaerji Scots Pine National Forest Park (Z2)	119.99°N 48.27°E	2.6	334.16
Uber Boliger (Z3)	119.34°N 47.55°E	1.32	292.08
Daqinggou National Nature Reserve (Z4)	122.18°N 42.82°E	7.56	465.82
Baiyin Aobao National Nature Reserve (Z5)	119.14°N 43.50°E	3.84	379.26
Huamugou National Forest Park (Z6)	116.47°N 42.29°E	3.84	379.26
Saihanwula National Nature Reserve (Z7)	118.20°N 43.70°E	6.68	362.08
Sumu Mountain Forest Park (Z8)	113.40°N 40.29°E	6.18	414.28
Manhan Mountain National Forest Park (Z9)	112.07°N 40.11°E	6.42	384.62
Haraqin Ecological Park (Z10)	111.73°N 40.90°E	7.32	432.84
EjinHoro Ten Thousand Mu Scots Pine Forest (Z11)	109.74°N 39.58°E	7.86	426.8
Yingpan Mountain Ecological Park (Z12)	105.72°N 38.19°E	9.5	253.36

### 2.2 Sample collection and processing

To thoroughly investigate the diversity of soil fungi in the Inner Mongolia region, soil samples were systematically collected from typical PSM forest areas along a continuous gradient stretching from east to west, encompassing Hulunbuir, Chifeng, Tongliao, Ulanqab, Hohhot, Ordos, and Alxa League.

A total of 12 sampling sites were established for this study, and each site underwent six replicate samplings to ensure the reliability of the data. At each site, six plots of 20 m × 20 m were selected in areas with minimal variation in topography and slope. These plots were spaced 50–100 m apart. Within each plot, three mid-aged trees with similar growth conditions were randomly selected, maintaining a minimum distance of 10 m between each tree. Sampling involved a five-point sampling method around the east, west, south, and north sides of the selected trees, within a soil depth of 20 cm. Soil samples from each plot were mixed to represent one replicate.

All collected samples were sieved through a 2 mm mesh. Each sample was then divided into two portions: one was stored at −80°C for subsequent high-throughput sequencing analysis. For the soil enzyme activity assays, each sampling site was replicated six times to ensure the reliability and reproducibility of the data. Soil enzyme activities were measured in each replicate sample to account for any spatial variability within the sampling plots. The assays were conducted on air-dried soil samples, and enzyme activities were quantified using ELISA kits. This replication allows for robust statistical analysis to validate the significance of differences observed in enzyme activities across the various sampling sites.

### 2.3 Methodology analysis and statistical data

The methodology used to explore the molecular diversity of soil fungal communities in PSM forests in Inner Mongolia employed a metagenomic sequencing approach, which included DNA extraction, library preparation, sequencing, and comprehensive bioinformatics analysis. Total genomic DNA was extracted from soil samples using the cetyltrimethylammonium bromide method. The quality of the extracted DNA, including degradation, contamination, and concentration, was rigorously assessed using the Agilent 4200 TapeStation system to ensure high-quality input material for subsequent sequencing.

Sequencing libraries were prepared using the NEBNext^®^ Ultra™ DNA Library Prep Kit for Illumina (NEB, USA, Catalog#: E7370L), following the manufacturer’s guidelines. Each sample was assigned a unique index for multiplexing. Genomic DNA was fragmented to an average size of 350 bp via sonication. The resulting DNA fragments underwent end-polishing and A-tailing before being ligated with full-length Illumina sequencing adapters. PCR amplification enriched the adapter-ligated fragments, which were then purified using the AMPure XP system (Beverly, USA). Library quality was evaluated on the Agilent 5400 system (Agilent, USA) and quantified by QPCR (1.5 nM). Qualified libraries were pooled based on effective concentration and the desired data output, then sequenced on the Illumina NovaSeq 6000 platform using a Pair-End 150 bp strategy. The Illumina NovaSeq 6000, a state-of-the-art next-generation sequencing technology, ensured high throughput and accuracy, generating approximately 6 Gb of raw data per sample through its sequencing-by-synthesis approach.

Raw metagenomic sequencing data underwent thorough preprocessing using KneadData software to ensure data reliability. Quality control involved the use of Trimmomatic to remove sequencing adapters, low-quality bases, and sequences shorter than 50 bp. To address potential host DNA contamination, Bowtie2 (https://bowtie-bio.sourceforge.net/bowtie2/) was employed to filter out host-derived reads by aligning the cleaned data against a host reference database, ensuring that subsequent analyses focused exclusively on microbial communities. Finally, FastQC was used to assess the effectiveness of these quality control measures by evaluating data quality before and after trimming.

Species composition was determined by aligning the quality-controlled sequencing reads to a comprehensive microbial nucleic acid database. This database was constructed from fungal sequences selected from the NCBI NT nucleic database and the RefSeq whole-genome database. Kraken2, a powerful tool for rapid taxonomic classification of metagenomic sequences, was used to assign taxonomic labels to the reads based on this reference database, enabling precise identification of fungal species in the soil samples. Following the initial taxonomic assignment, the abundance of species in the samples was estimated using Bracken (Bayesian Reestimation of Abundance after Classification), which refined Kraken2’s initial abundance estimates by correcting for biases in read classification through Bayesian inference. After species annotation, the community composition of the samples was statistically analyzed at various taxonomic levels: kingdom, phylum, class, order, family, genus, and species.

Further analysis of high-throughput sequencing data was performed using the Microbiome Union Bioinformatics Cloud platform (https://www.bioincloud.tech/). The platform was used to generate percentage-stacked bar charts illustrating the relative abundance of soil fungi and *Suillu*s species. Additionally, α diversity indices, such as Chao1 and Shannon indices, were calculated at the genus level to assess fungal richness and diversity across different forest sites. To assess the variation in fungal community diversity across different sampling sites, statistical tests were conducted using one-way analysis of variance to compare α diversity indices, including Chao1 and Shannon indices, between sites. *Post-hoc* Tukey’s honestly significant difference test was applied to identify specific pairs of sites with significant differences. The results were considered statistically significant at *p-*values <0.05. This statistical approach ensured that the observed differences in diversity were not due to random variation and were robust to environmental gradients.

### 2.4 Soil enzyme activity assays

Soil enzyme activity has become an essential metric for quantifying ecosystem functions, serving as an indicator of soil quality and functionality. In this experiment, soil enzyme activities were determined using ELISA kits, with the absorbance of samples measured at 450 nm using a multi-function enzyme reader. The enzymes analyzed included urease, phosphatase, β-d-glucosidase (β-glu), β-xylosidase (β-xyl), cellulase (CBH), peroxidase, protease, β-*N*-acetyl-glucosaminidase, and leucine aminopeptidase (LAP).

### 2.5 Data acquisition

The sequencing data involved in this study have been uploaded to the NCBI database (https://www.ncbi.nlm.nih.gov/) under the accession number PRJNA1095633.

## 3 Results and analysis

### 3.1 Diversity analysis of PSM soil fungal communities

High-throughput sequencing technology was employed to analyze the structure of fungal communities in PSM soil samples from the Inner Mongolia region. At a 97% sequence similarity level, clustering of operational taxonomic units (OTUs) identified a total of 841 fungal OTUs. These OTUs were classified into 9 fungal phyla, 33 classes, 94 orders, 211 families, 401 genera, and 795 species.

**Figure 2:**
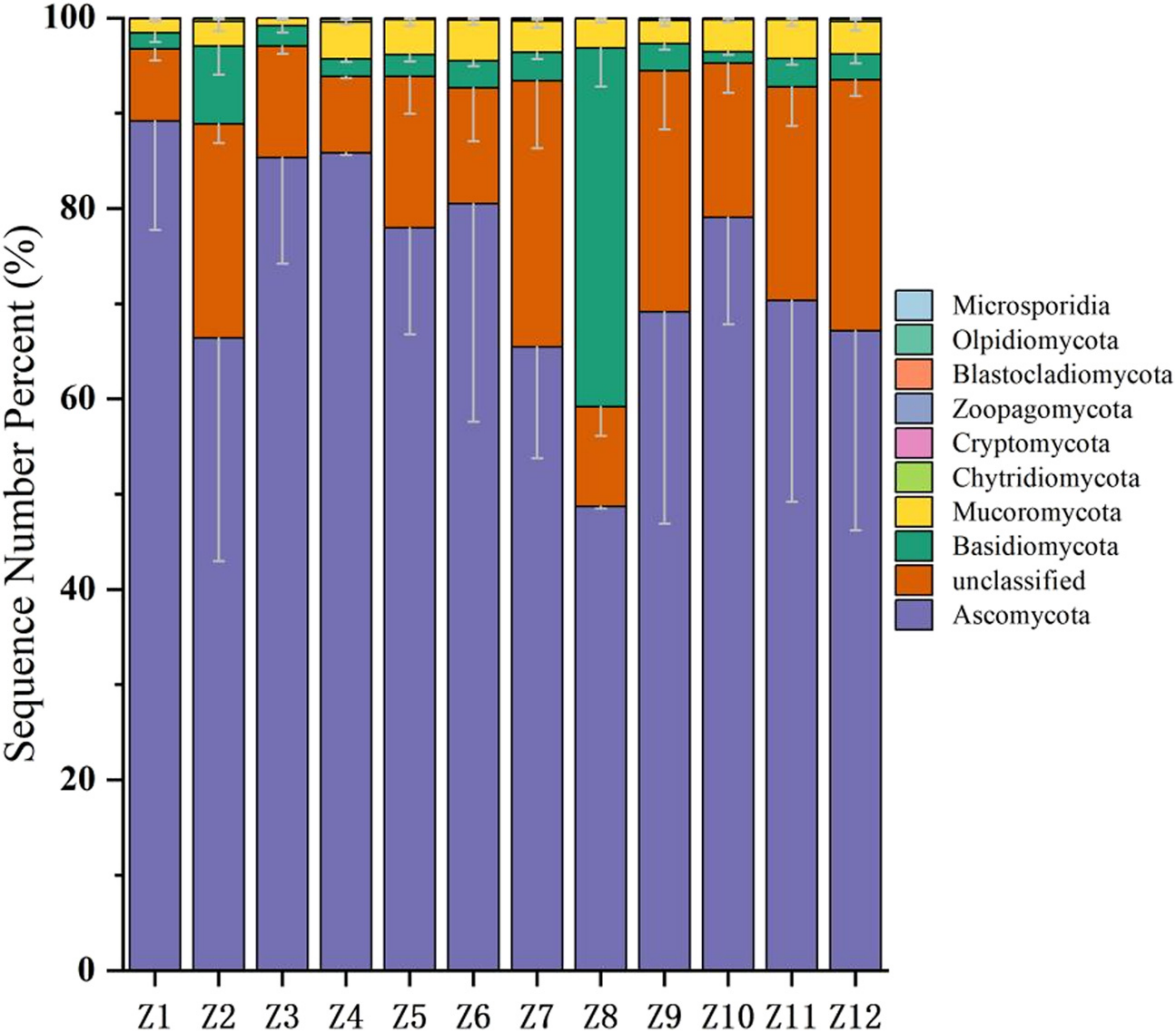
Phylum-level species composition of soil fungi in PSM forests in Inner Mongolia. Note: The color of the column represented the abundance of the abundance gate level, and the error line (the vertical line at the top of the stacking part of each taxonomic unit) was added to represent mean ± standard deviation (SD), reflecting the variability of biological repetition within the group (*n* = 6).

In the phylum-level classification of the PSM soil fungal community structure (Figure 2), nine phyla were detected, including Ascomycota, Basidiomycota, Mucoromycota, Chytridiomycota, Cryptomycota, Zoopagomycota, Blastocladiomycota, Olpidiomycota, and Microsporidia. The three dominant fungal phyla in the Inner Mongolia region varied, with ranges of 55.85−99.00, 2–42.5, and 0.9–4.1%, respectively. Except for the Sumu Mountain Forest Park (Z8), where Basidiomycota was the dominant phylum, Ascomycota was the predominant phylum in other areas.

**Figure 3:**
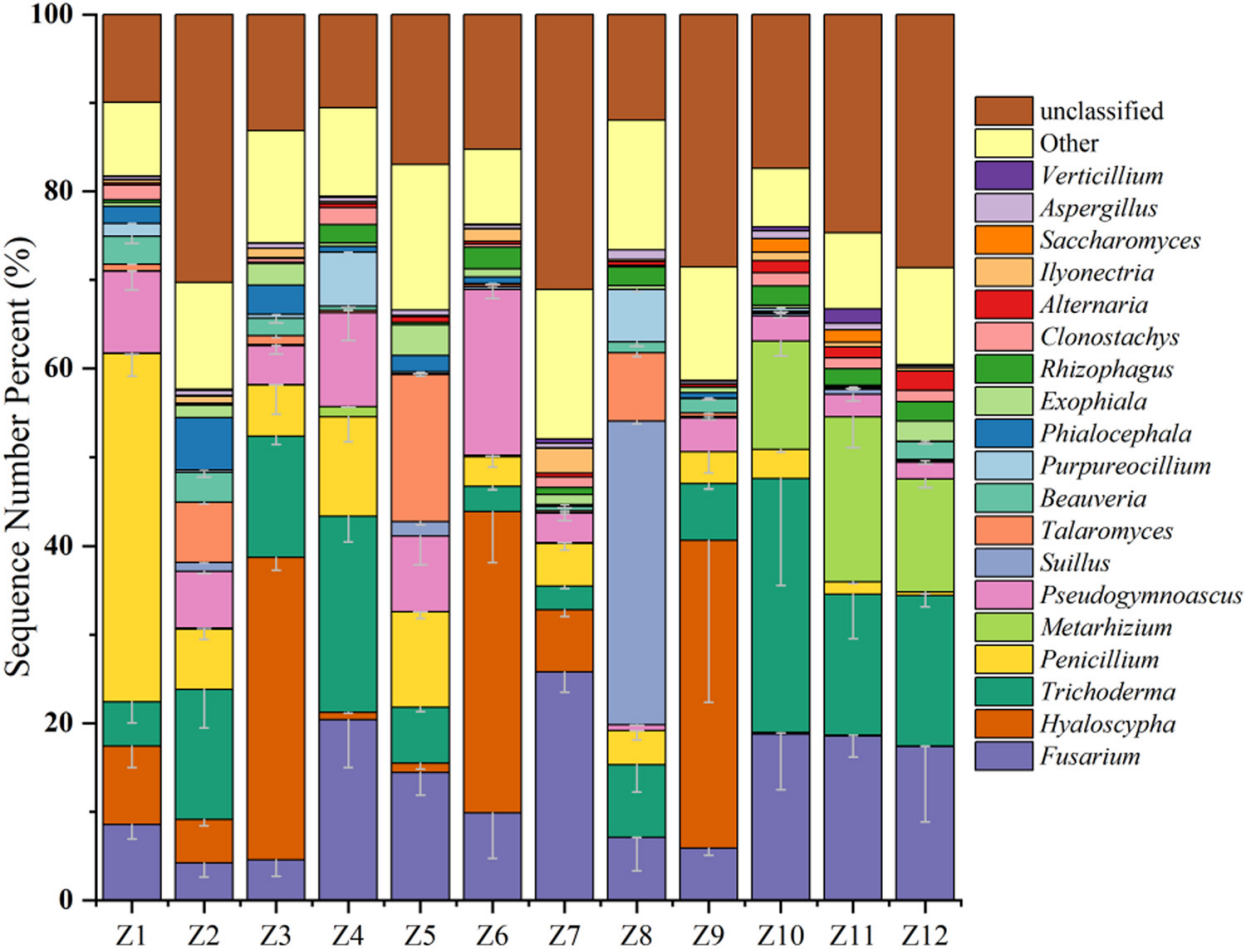
Genus-level species composition of soil fungi in PSM forests in Inner Mongolia. Note: The color of the cylinder represents the top 20 genera in abundance, and the top 10 genera in abundance add error lines (vertical lines at the top of the stacked parts of each taxon), indicating mean ± SD, reflecting the variability of biological repetition within the group (*n* = 6).

In the genus-level classification of the PSM soil fungal community structure (Figure 3), the ten most abundant dominant genera were *Fusarium*, *Metarhizium*, *Penicillium*, *Trichoderma*, *Hyaloscypha*, *Suillus*, *Pseudogymnoascus*, *Talaromyces*, *Purpureocillium*, and *Beauveria*. Geographically, the relative abundance of *Penicillium* and *Hyaloscypha* showed a decreasing trend from east to west across Inner Mongolia, while the relative abundance of *Fusarium*, *Metarhizium*, and *Beauveria* exhibited an increasing trend from east to west. In the Harachin Ecological Park (Z10), the Yijinholo Banner’s Ten Thousand Mu PSM Forests (Z11), and the Yingpan Mountain Ecological Park in Alxa Left Banner (Z12), the fungi *Pseudogymnoascus*, *Talaromyces*, and *Purpureocillium* showed lower relative abundances; whereas *Trichoderma* had the lowest relative abundance in the Baiyin Aobao National Nature Reserve (Z5) and the Huamugou National Forest Park (Z6).

### 3.2 Distribution patterns of *Suillus* fungi in PSM soils

**Figure 4:**
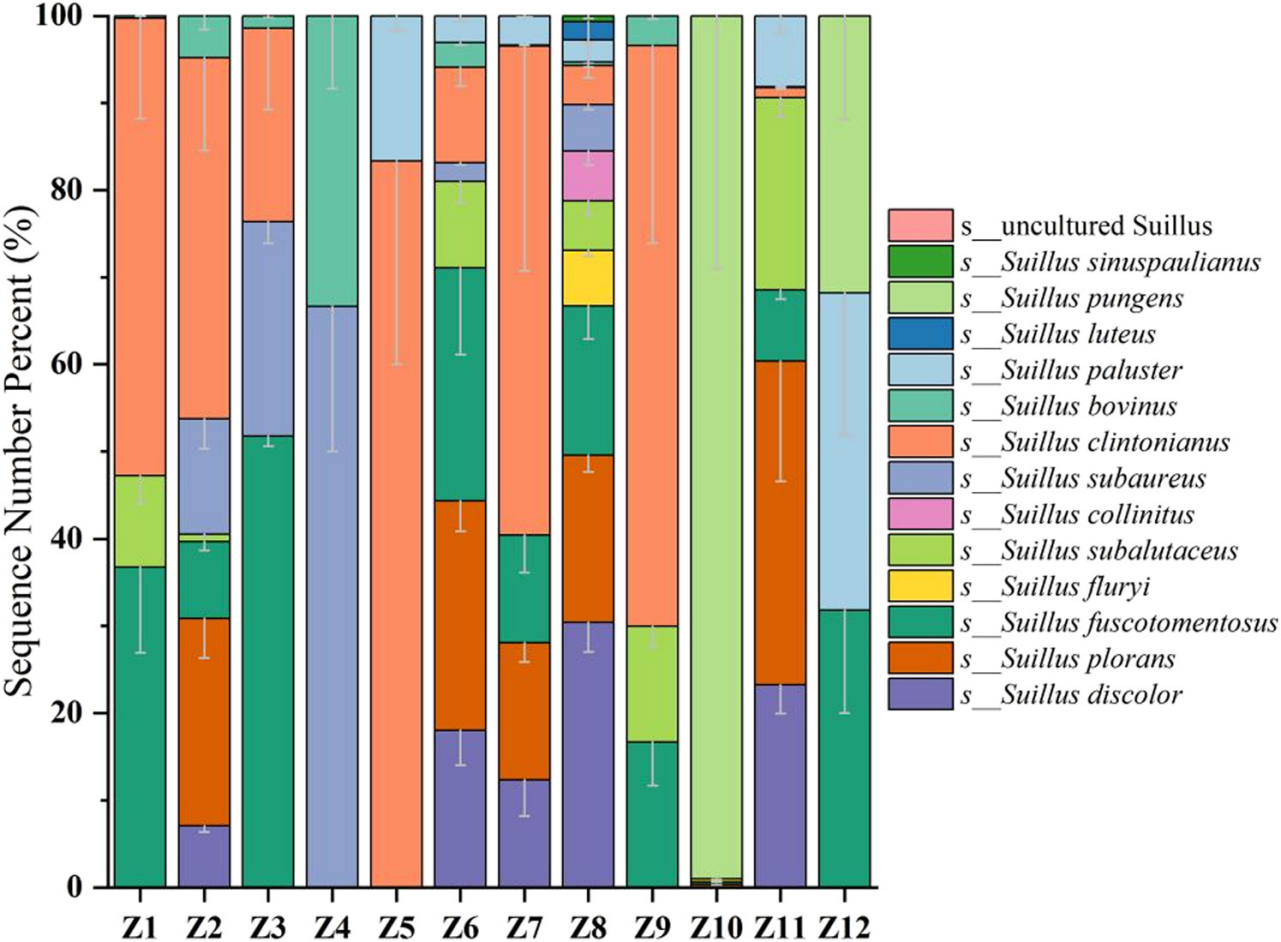
Distribution patterns of *Suillus* species in PSM forest soils across Inner Mongolia. Note: All color blocks in the figure represent all *Suillus* species detected, and all species add error lines (vertical lines at the top of the stacked parts of each taxon), indicating mean ± SD, reflecting the biological repetitive variability within the group (*n* = 6).

At the species taxonomic level (Figure 4), 13 species of *Suillus* fungi were detected in PSM soil samples, including *S. bovinus*, *S. paluster*, *S. subalutaceus*, *S. subaureus*, *S. fluryi*, *S. plorans*, *S. clintonianus*, *S. discolor*, *S. fuscotomentosus*, *S. collinitus*, *S. luteu*s, *S. pungens*, and *S. sinuspaulianus*. Among the 13 *Suillus* species detected, *S. clintonianus* was the dominant species in all regions, with relative abundances ranging from 29.29 to 66.67%. This consistent dominance across the study area suggests that *S. clintonianus* may possess traits that confer competitive or environmental advantages, such as higher infection rates with Pinaceae and the ability to thrive in nutrient-poor soils. These traits may allow *S. clintonianus* to outcompete other species in this semi-arid environment, where water and nutrient availability are limiting factors. Future studies should explore whether *S. clintonianus* exhibits specific physiological or biochemical adaptations, such as drought tolerance or enhanced nutrient uptake mechanisms.

The distribution patterns of *Suillus* in PSM soils (Figure 4) revealed that 8 *Suillus* species were detected in the soils of the Hulunbuir region (Z1, Z2, and Z3); 2 species were found in the Tongliao area (Z4); 8 species were identified in the Chifeng region (Z5, Z6, and Z7); and 12 species were observed in the Ulanqab region (Z8 and Z9). *S. clintonianus* was a dominant species in all the above regions, with its highest proportion reaching 66.67%. In the PSM forest soils of the Hohhot area (Z10) and Alxa region (Z12), three *Suillus* species were detected. In these areas, *S. subalutaceus* (60%) and *S. fuscotomentosus* (53.13%) were the dominant species in Z10 and Z12, respectively, as well as in the Ten Thousand Mu PSM Forest of Yijinholo Banner (Z11).

### 3.3 Diversity analysis of fungal communities in PSM forests

**Figure 5:**
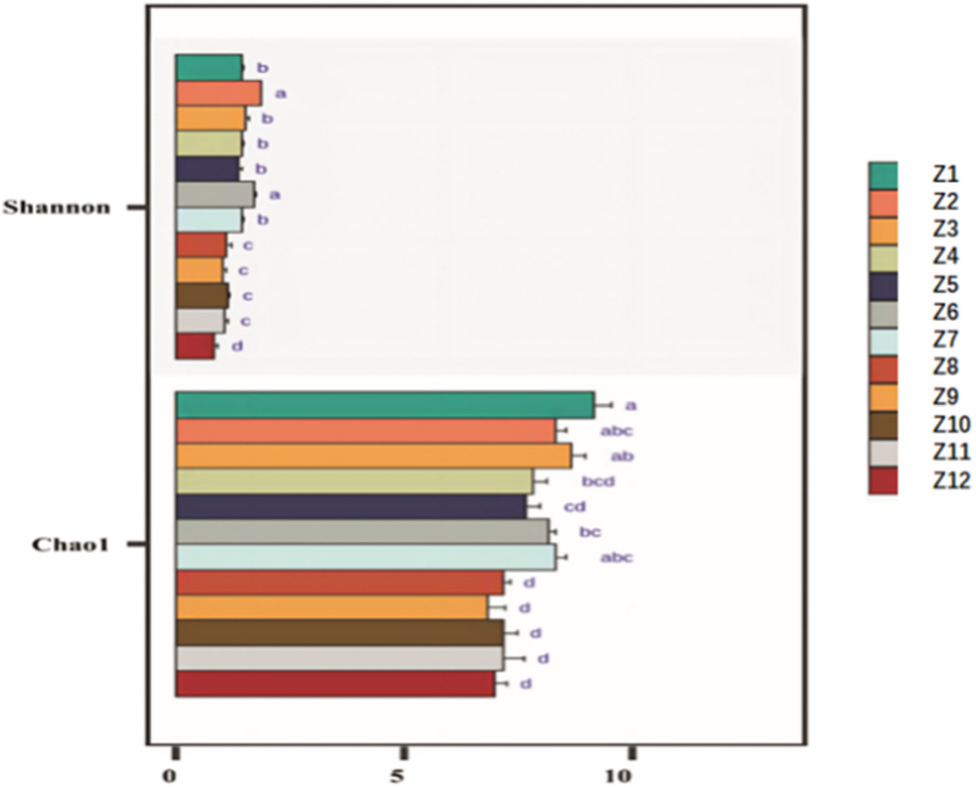
α Diversity of soil fungi in PSM forests in Inner Mongolia.

The diversity and richness of fungal communities in the PSM forest soils of the Inner Mongolia region exhibit significant geographical gradients. Specifically, within the study area extending from east to west, both the Shannon diversity index and the Chao 1 richness index show a gradual decreasing trend (Figure 5). These results suggest that the diversity and richness of soil fungal communities may be influenced by geographical and environmental factors in their spatial distribution.

**Figure 6:**
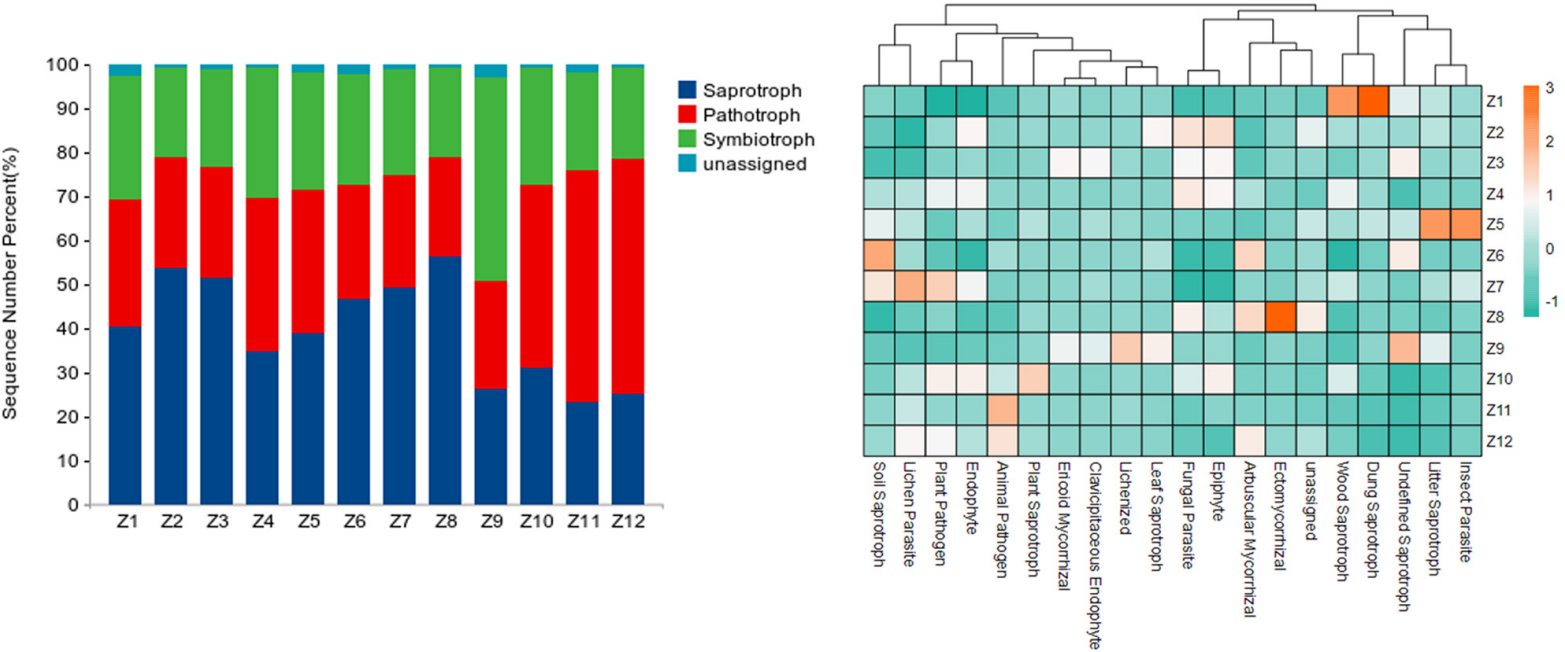
Proportions of trophic modes and heat map of relative abundance of fungal functional groups.

Three primary trophic types are represented across 18 functional groups (Figure 6), with the top five abundant groups being animal pathogens, wood saprotrophs, undefined saprotrophs, plant pathogens, and endophytes. Among the symbiotic trophic types, ECM fungi show high abundance, particularly peaking in the Z8 area. Within the pathotrophic category, animal pathogens comprise the highest proportion, displaying an increasing trend from east to west. Plant pathogens reach their highest abundance in the Z7 area, showing no significant pattern from east to west.

Among the saprotrophic types, wood saprotrophs dominate and exhibit a decreasing trend from the eastern to the western parts of Inner Mongolia. The rhizosphere soil fungal functional groups of PSM forests undergo changes, with variations in the abundance of the 18 functional groups across 12 typical PSM forest sites. This indicates that the main functions performed by the fungal communities vary across these locations.

### 3.4 Correlation of *Suillus* with soil enzyme activities and meteorological factors

**Figure 7:**
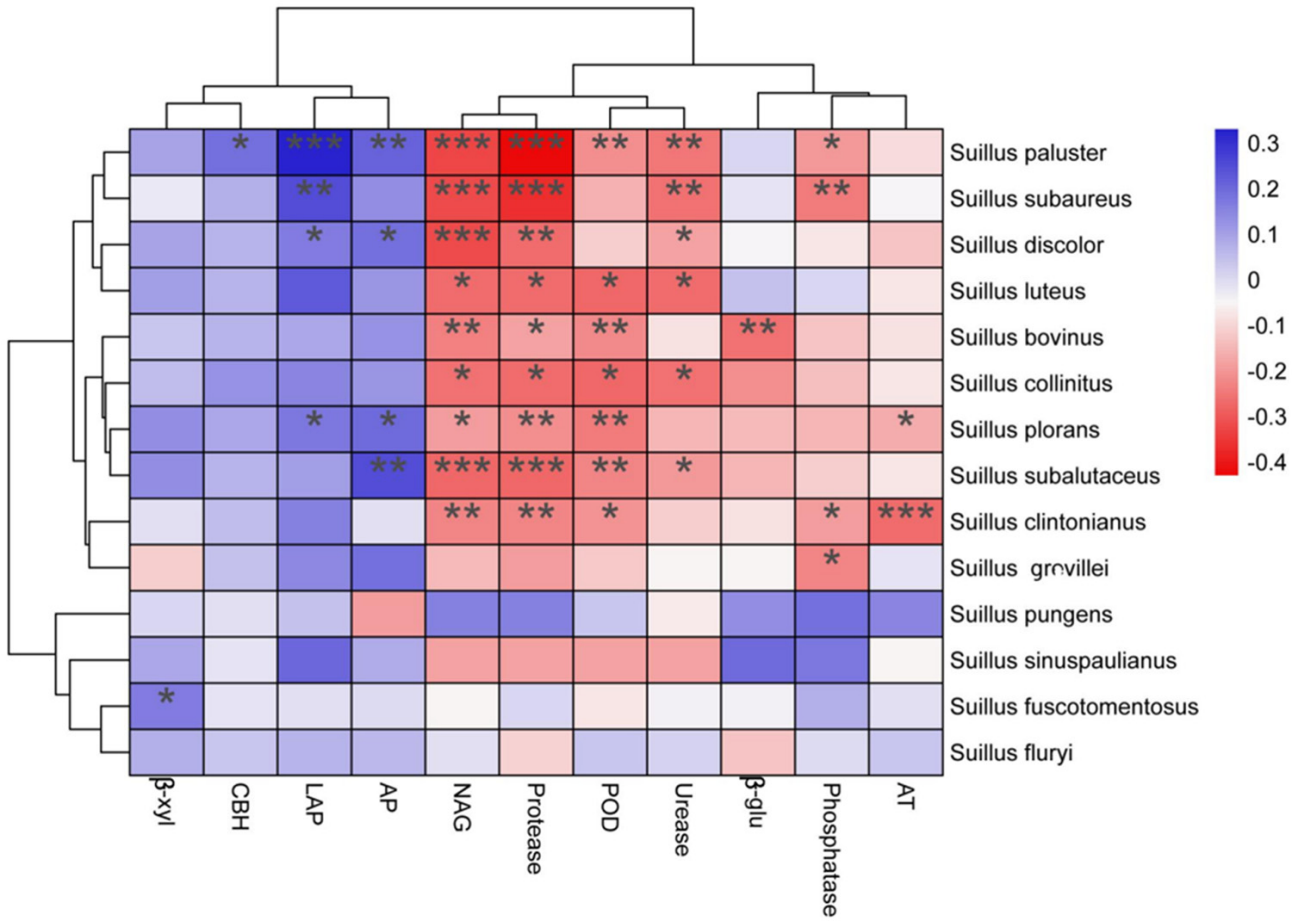
Correlation analysis of *Suillus* with soil enzyme activities and meteorological factors.

A correlation analysis was conducted between all detected *Suillus* species in PSM forest soils and both soil enzyme activities and meteorological factors (Figure 7). The results revealed a highly significant positive correlation between soil LAP and the species *S. paluster*, *S. subaureus*, *S. plorans*, and *S. discolor*. Significant positive correlations were also observed between soil β-xyl and *S. fuscotomentosus*, and between soil CBH and *S. paluster*. Furthermore, AP exhibited a highly significant positive correlation with *S. paluster*, *S. subalutaceus*, *S. discolor*, and *S. plorans*. Overall, the *Suillus* species are primarily influenced by precipitation and soil LAP enzyme activity.

## 4 Conclusions and discussion

While this study provides valuable insights into the diversity and distribution of *Suillus* fungi in PSM forests. Long-term monitoring across multiple seasons would provide a more comprehensive understanding of how *Suillus* and other fungal communities fluctuate over time, particularly in response to seasonal changes in precipitation and temperature. Future research should also investigate the role of *Suillus* in promoting forest health under varying climatic conditions and explore its potential for enhancing forest resilience to climate change. Soil fungal diversity serves as a critical indicator of soil quality, vividly reflecting the dynamic characteristics of fungal communities [11–13]. The study identified several key environmental factors that significantly impact the structure of soil fungal communities, including soil pH, temperature, moisture content, total nitrogen, ammonium nitrogen, total carbon, and enzyme activities. These environmental drivers not only shape fungal community composition but also have important implications for forest health and management. For instance, changes in precipitation patterns and soil moisture content could influence the abundance of ECM fungi such as *Suillus*, which play a crucial role in enhancing tree growth and forest resilience. Forest management practices should consider these factors, such as by monitoring soil moisture and nutrient content, which can help inform strategies to improve forest health under changing climatic conditions [14].

The observed east-to-west gradient in fungal diversity, with decreasing *Suillus* richness and increasing *Fusarium* abundance from east to west, reflects an environmental gradient driven by changing precipitation and soil nutrient availability. This spatial gradient suggests that regions with lower *Suillus* richness may be more vulnerable to soil degradation and fungal pathogens, such as *Fusarium*, which can negatively affect forest health. For forest management and climate adaptation strategies, these patterns highlight the need for region-specific interventions. Areas with low *Suillus* richness and high *Fusarium* abundance could benefit from targeted fungal inoculation to restore microbial balance and enhance forest resilience. Inoculating *Suillus* species in these regions could help improve nitrogen availability, suppress *Fusarium* pathogens, and promote a more balanced soil microbial community. Such interventions could be crucial for enhancing soil fertility and improving tree growth in areas facing environmental stress due to climate change [15].

Given these patterns, region-specific forest management strategies should be considered. In areas where *Suillus* richness is low and *Fusarium* abundance is high, targeted fungal inoculation with *Suillus* species could help improve soil microbial balance by enhancing nitrogen availability and suppressing the growth of *Fusarium* pathogens. Inoculation with *Suillus* fungi has been shown to promote plant growth by enhancing nutrient uptake and reducing the negative effects of soilborne pathogens. Additionally, soil amendments, such as the addition of organic matter or biofertilizers, could support the establishment of *Suillus* and other beneficial ECM fungi, further improving soil quality and forest resilience. These targeted interventions could contribute to reforestation and restoration efforts in semi-arid regions, where forest health is at risk due to environmental stressors.

Similar studies in boreal and temperate forests have shown that a single ECM fungus, such as *Suillus*, can significantly improve tree growth and health by facilitating nutrient uptake. This study in PSM forests aligns with these findings but adds a novel contribution by highlighting the distinct geographic and environmental factors that influence *Suillus* distribution and its interactions with other fungal communities in semi-arid climates [16]. After inoculation with *S. luteu*s, the abundance of *Fusarium* in the rhizosphere soil samples from pines decreased, suggesting that inoculation with *Suillus* fungi suppressed *Fusarium*, shifting the dominance to *Suillus* and enhancing soil fungal diversity. Inoculation with *S. luteus* also altered the bacterial community structure in the rhizosphere and enhanced nutrient availability in the soil. These results indicate that beneficial fungi can suppress pathogen invasion to some extent, thereby promoting plant growth [17].

This study found that when beneficial fungi like *Suillus* and *Trichoderma* are abundant, the abundance of *Fusarium* pathogens is reduced. It was discovered that inoculation with *Suillus* significantly enhances the resistance of PSM seedlings to dieback disease, possibly because *Suillus* effectively increases the concentration of chlorophyll, activities of polyphenol oxidase, and superoxide dismutase in PSM, reduces the disease index of PSM dieback, and the activity of catalase, thereby inhibiting the growth of some pathogenic fungi [18–20].

ECM fungi play a vital role in promoting tree growth, enhancing trees’ ability to absorb mineral nutrients, increasing trees’ resistance to adverse conditions, and maintaining forest ecosystem stability [21]. Specifically, *Suillus* with its high infection rate in Pinaceae and significant impact on endogenous hormone levels during symbiosis, plays a key role in directly or indirectly regulating the physiological processes of symbiotic plants [22]. Additionally, *Suillus* combined with plant growth-promoting bacteria (PGPR) can enhance plant growth, alter the physicochemical properties, enzyme activities, and microbial community structure of plant rhizosphere soil. *Suillus* species exhibit remarkable tolerance to heavy metals, particularly in environments with elevated soil contamination. This trait is believed to be linked to the ability of *Suillus* to sequester heavy metals in fungal tissues, thus reducing the bioavailability of these metals to plants and other soil organisms [23]. This tolerance is thought to be a key mechanism by which *Suillus* can thrive in disturbed soils, making it a valuable tool for bioremediation. The application of *Suillus* in ecological restoration could enhance the rehabilitation of degraded lands by improving soil quality and promoting the re-establishment of vegetation in metal-contaminated environments. Further studies on the molecular mechanisms behind this tolerance, including the role of extracellular enzymes and metal-binding proteins, are warranted to fully understand *Suillus*’ potential in environmental management [24].

Similar studies in temperate and boreal forests have demonstrated the critical role of *Suillus* species in promoting tree health and nutrient cycling [25]. The findings of this study align with these results, highlighting the adaptability of *Suillus* to semi-arid environments and its potential to support forest resilience under changing climatic conditions. *Suillus* species, such as *S. clintonianus*, which dominate in Inner Mongolia’s PSM forests, exhibit broad adaptability to varying environmental conditions, further underscoring the ecological flexibility of *Suillus* species in supporting forest ecosystems across different climates [26].

*Suillus* fungi exhibit broad adaptability and low environmental dependency, traits that are particularly evident in their ability to colonize diverse forest ecosystems. Similar studies in temperate and boreal forests have shown that *Suillus* species, such as *S. luteus* and *S. bovinus*, play critical roles in nutrient cycling and enhancing plant growth, especially under nutrient-limited conditions. The results of this study, which show the dominance and adaptability of *S. clintonianus* in semi-arid conditions, further highlight the ecological flexibility of *Suillus* species and their potential to support forest health across various climatic zones. In this study, 13 species of *Suillu*s fungi were identified from the PSM forests soils in Inner Mongolia. The distribution of *Suillus* shows significant geographical variation, with the abundance of *Suillus* in the Chifeng area being higher than in other regions. This study found that the diversity and richness of *Suillus* fungi under PSM forests increase along an east-to-west geographical gradient.

The results of this study show that fungal communities in PSM forests adapt to environmental changes by adopting various nutritional strategies, including saprotrophic, pathogenic, and symbiotic modes. The predominance of saprotrophic fungi, which play a key role in nutrient cycling, suggests that these fungi are critical for maintaining soil fertility in nutrient-poor environments. The high abundance of ECM fungi like *Suillus* underscores their importance in enhancing plant growth, promoting nutrient uptake, and improving forest resilience to environmental stressors. In this study, the main nutritional types in different PSM forests were saprotrophic, pathogenic, and symbiotic fungi, with a predominance of saprotrophic fungi in PSM forest soils, whose main role is to decompose organic matter in the soil into mineral nutrients absorbable by plants. Ascomycetes, mostly saprotrophs, can decompose a variety of recalcitrant substances, playing a crucial role in the decomposition of organic matter in the soil and being insensitive to environmental stress. The high proportion of saprotrophic fungi in the soils may be related to the PSM litter providing nutrients for the associated microbial communities. Plant pathogenic fungi occupy a middle proportion among all functional groups, with no apparent trend in their distribution across the PSM forests of Inner Mongolia. The proportion of symbiotic nutritional types is low, but the abundance of ECM functional groups is high. ECM fungi are significant in promoting the decomposition of organic and inorganic elements in the soil, enhancing plant disease resistance and stress resistance. Basidiomycetes are commonly found ECM fungi that can degrade substances like lignin that are difficult to decompose, promoting nutrient cycling in forest soils [27,28].

Soil enzymes play a crucial role in the material cycling and energy flow of soil ecosystems. The strong correlations observed between LAP activity and the distribution of *Suillus* species suggest a significant role of this enzyme in nitrogen cycling, particularly in nitrogen-limited environments. LAP, an enzyme involved in breaking down proteins and peptides into bioavailable nitrogen, directly influences the nitrogen availability in soil. In *Suillus*, increased LAP activity likely enhances the nitrogen supply, supporting the establishment and growth of ECM fungi. This is especially important in semi-arid regions, where nitrogen scarcity is a key limiting factor for plant growth. By enhancing nitrogen availability, *Suillus* can improve tree growth and forest resilience, particularly under drought stress, where nitrogen is further limited. The role of LAP in supporting *Suillus* species competitiveness in nutrient-poor soils could explain their dominance in semi-arid forest ecosystems and highlight the crucial role of *Suillus* in improving nutrient cycling and forest health under environmental stress [13]. The distribution of *Suillus* species is strongly correlated with environmental variables, particularly precipitation and LAP activity. These correlations suggest that *Suillus* species are well-adapted to water-limited environments, where the ability to mobilize nitrogen through LAP activity provides a competitive advantage. In semi-arid ecosystems, *Suillus* plays a critical role in enhancing nutrient availability through its ECM relationships with coniferous hosts, particularly in nitrogen-poor soils. This ability to increase nitrogen uptake under drought conditions contributes to tree resilience and supports the ecological stability of forest ecosystems. *Suillus*’ role in nutrient cycling and stress tolerance, especially under fluctuating precipitation, further underscores its importance in maintaining forest health, particularly in the face of climate change [20]. Future research should focus on understanding the long-term effects of environmental changes on fungal community dynamics, particularly in relation to *Suillus* species. Long-term studies that monitor *Suillus* populations over multiple seasons would provide a more comprehensive understanding of how these fungi respond to seasonal fluctuations in precipitation and temperature. Experimental studies could also explore the potential of *Suillus* inoculation as a strategy for improving forest health in semi-arid regions, especially under climate change scenarios. Moreover, further research on the interactions between *Suillus* and other microbial communities, such as PGPR, could provide valuable insights into how these symbiotic relationships contribute to forest resilience. In-depth studies on such fungi, especially the ecological functions and application potential of *Suillus*, are crucial for exploring and utilizing this biological resource to promote the forest economy of Inner Mongolia.
